# Neuropsychiatric Side Effects of Hydroxychloroquine in a Patient With Idiopathic Pulmonary Hemosiderosis

**DOI:** 10.1111/jpc.70001

**Published:** 2025-02-05

**Authors:** Didar Ağca Cengiz, Abdurrahman Erdem Başaran, Betül Bankoğlu Parlak, Irmak Tanal Şambel, Ayşen Bingöl

**Affiliations:** ^1^ Department of Pediatric Pulmonology Akdeniz University Faculty of Medicine Antalya Turkey

**Keywords:** case report, hydroxychloroquine, neurology, pharmacology, respiratory, side effect

## Abstract

**Background:**

Idiopathic pulmonary hemosiderosis (IPH) is a rare interstitial lung disease. Glucocorticosteroids and hydroxychloroquine are the most commonly used treatments. Although neuropsychiatric side effects related to hydroxychloroquine use are seen in adult cases, only one paediatric patient has been reported in the literature.

**Case Presentation:**

We report a case of a 6‐year‐old girl with IPH, who developed neuropsychiatric symptoms, including restlessness, confusion and myoclonic movements, after the therapeutic use of hydroxychloroquine.

**Conclusion:**

With increasing knowledge and experience of interstitial lung disease, the use of hydroxychloroquine treatment is increasing. It is important to remember that hydroxychloroquine is a central nervous system stimulant, and neuropsychiatric side effects may be seen in children. This report highlights the importance of recognising potential neuropsychiatric side effects in paediatric patients using hydroxychloroquine, especially when combined with corticosteroids or other risk factors.

**Summary**

- Idiopathic pulmonary hemosiderosis (IPH) is a rare lung disease treated primarily with glucocorticosteroids and hydroxychloroquine, which is increasingly used for its immunomodulatory effects.
- Hydroxychloroquine, despite its wide use, can cross the blood–brain barrier and cause neuropsychiatric side effects, even at recommended therapeutic doses.
- Hydroxychloroquine must be closely monitored for neuropsychiatric adverse effects, especially when co‐administered with steroids, CYP3A4 inhibitors, or other medications that may enhance its central nervous system penetration and side effects.

## 1 Introduction

Idiopathic pulmonary hemosiderosis (IPH) is a rare disease diagnosed after exclusion of other causes of diffuse alveolar haemorrhage. Although the pathophysiology of the disease is not fully understood, it is thought to have an immunological basis [1]. Although corticosteroids are the first‐line treatment, immunomodulatory/immunosuppressive agents such as hydroxychloroquine, azathioprine and cyclophosphamide are used in refractory disease [2]. Hydroxychloroquine is an antimalarial drug with an immunomodulatory effect that is widely used in autoimmune diseases. It is one of the most preferred non‐steroidal treatments for IPH patients due to its low side effect profile when used with an immunomodulatory treatment dose [3, 4, 5]. We report here a paediatric case of a 6 years old female patient with neuropsychiatric symptoms following the use of therapeutic doses of hydroxychloroquine for IPH.

## 2 Case Presentation

**FIGURE 1:**
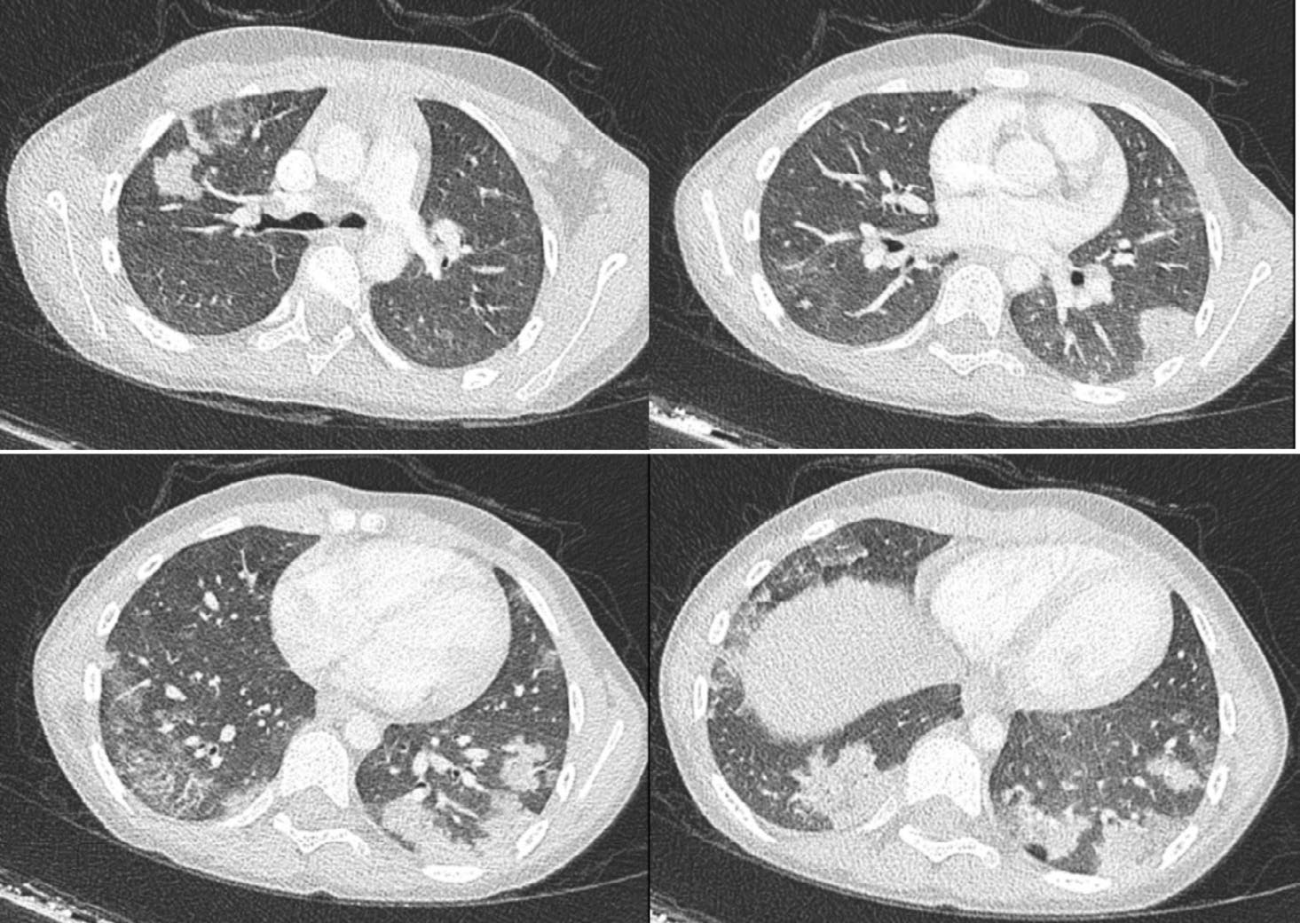
Patchy consolidated areas and accompanying ground glass areas in both lungs.

A 6‐year‐old girl applied to the emergency department with the complaint of hemoptysis following a wet cough of about 10 days duration. The patient had been seen in the paediatric nephrology department for 2 months with findings of proteinuria. After renal biopsy, she was diagnosed with focal segmental glomerulosclerosis (FSGS) and was being treated with enalapril. She had no other illness or history of neuropsychiatric disease other than FSGS. In her family history, there was no consanguinity between the parents and no history of neuropsychiatric disease. On physical examination, the patient's saturation was 98% on room air and her respiratory rate was 22 breaths per minute. Her vitals were all within normal limits, and there was no pathology in her lung sounds and other system examinations. Her weight was 25 kg (73th centile), height was 123 cm (70th centile) and BMI was 16.5 kg/m^2^ (69th centile). Acute phase reactants and basic metabolic panel were all within normal limits. Haemoglobin value was 7.4 g/dL, leukocyte count was 12.300/mm3 and neutrophil count was 7600/mm3. Coagulation parameters were within normal limits. The patient was admitted to the hospital with ceftriaxone treatment and further tests were performed. Chest computed tomography (CT) scan showed patchy consolidations and associated ground‐glass opacities in both lungs (Figure 1). The present findings were investigated for infectious causes, pulmonary vasculitic processes and other causes of diffuse alveolar haemorrhage. Tuberculosis was excluded with a negative PPD result. Anti‐GBM antibody was negative, cow milk specific IgG was negative, ANA was negative, ANCA was negative, C3 and C4 levels were normal. Tissue transglutaminase IgG and IgA were negative. The patient underwent a bronchoscopy and the anatomical structure of the respiratory tract was observed normally and no specific findings were found. Bronchoalveolar lavage (BAL) tracheal, fungal and tuberculosis culture results were negative. Cytological examination revealed 95% haemosiderin‐laden macrophages. On the basis of these findings, the patient was diagnosed with idiopathic pulmonary haemosiderosis and was started on methylprednisolone at 2 mg/kg/g.

As the symptoms recurred during follow‐up with glucocorticosteroid treatment, the patient was started on 50 mg hydroxychloroquine divided in to two doses (2 mg/kg/d), and while the steroid dose was gradually reduced, the hydroxychloroquine dose was increased to 100 mg/day (4 mg/kg/d). In the second month of treatment, the patient was hospitalised due to recurrence of hemoptysis presenting with clinical features of bronchopneumonia. Treatment was adjusted to methylprednisolone 2 mg/kg/d and hydroxychloroquine 5 mg/kg/d. Clarithromycin (400 mg/day, 15 mg/kg/d) and ceftriaxone (1900 mg/day, 75 mg/kg/d) were started as antimicrobial treatment. The patient's hemoptysis regressed under the current treatments, but restlessness and agitation started on the 4th day of treatment.

A few hours later, neurological symptoms were observed, including the inability to recognise family members, double vision and suspicious myoclonic beats in the left extremities. The patient's symptoms regressed after benzodiazepine administration. Fingerstick blood glucose, serum glucose and electrolyte level were normal. Brain CT angiography, brain MRI and EEG were resulted as normal. The naranjo adverse drug reaction probability scale is a 10 item scale used to evaluate the likelihood of drugs causing adverse reactions [6]. It was administered and evaluated as a probable drug‐related reaction with a score of 6. Hydroxychloroquine treatment was discontinued and all findings improved in the 24th hour of follow‐up. The patient whose treatment was changed to steroid and azathiopurine is still being followed up and neuropsychiatric findings did not recur in 8 months under the current treatment.

## 3 Discussion

Interstitial lung disease is a group of lung diseases characterised by diffuse infiltration and irregular gas exchange, which are extremely rare in the paediatric population [7]. Clinical trials of treatments for these chronic conditions are limited. In case reports and a few retrospective series, hydroxychloroquine or chloroquine were the most commonly used agents in combination with glucocorticosteroid treatments [8]. The chILD‐EU protocol also recommends the use of hydroxychloroquine in addition to glucocorticosteroids [9].

IPH is a rare interstitial lung disease characterised by hemoptysis, radiologically diffuse infiltration and iron deficiency anaemia and diagnosed by exclusion of other causes of diffuse alveolar haemorrhage [10]. As in other interstitial lung diseases, the most commonly used agents are glucocorticosteroids and hydroxychloroquine. In a survey of 274 specialists from different countries following 274 cases of IPD, corticosteroids were reported to be the most commonly used drug at initial presentation and for maintenance treatment, and hydroxychloroquine was found to be the most commonly used drug in combination with corticosteroid therapy at initial presentation (33.3%) and for maintenance treatment (%64) [11].

Hydroxychloroquine belongs to the group of drugs known as 4‐aminoquinolines. It has a large volume of distribution and an associated long half‐life [12]. Following oral ingestion, blood level monitoring is approximately 0.8 h and the half‐life is as long as 40–60 days [13, 14]. Hydroxychloroquine crosses the blood–brain barrier and produces tissue concentrations 10–20 times higher than plasma concentrations, predisposing to neuropsychiatric side effects. Neuropsychiatric effects may be observed even at therapeutic doses [15]. The most common psychiatric side effect is anxiety, but psychosis, insomnia, irritability and self‐harming behaviours may also be observed [15, 16, 17]. As risk factors; known history of psychiatric illness, family history of similar conditions, female gender, low body weight, alcohol intake, concurrent use of low dose glucocorticosteroids, use of CYP3A4 inhibitor medical treatment, use with doses above 6.5 mg/kg/g have been reported [18]. It has been observed that the side effects have completely disappeared within a few hours up to a few months after the discontinuation of the drug. Only one paediatric case of neuropsychiatric adverse events due to hydroxychloroquine use has been reported in the literature. A 17‐year‐old girl with systemic lupus erythematosus has been reported to have acute psychosis 28 days after taking a therapeutic dose of hydroxychloroquine [19]. Our case was the youngest patient with neuropsychiatric side effects due to hydroxychloroquine use reported in the literature. Concurrent steroid use, initiation of clarithromycin because of the clinical presentation and revision of the hydroxychloroquine dose by increasing it may have predisposed to the development of the present side effect. Clarithromycin, as a strong CYP3A4 inhibitor, can slow the metabolism of hydroxychloroquine, leading to elevated drug levels and potentially amplifying its neuropsychiatric side effects [20]. Following the discontinuation of hydroxychloroquine treatment, the patient's neuropsychiatric symptoms completely resolved. However, due to the risk of recurrence of neuropsychiatric findings, hydroxychloroquine was not reintroduced. The patient was started on immunosuppressive therapy with azathioprine, and no recurrence of hemoptysis was observed. In this case, the simultaneous use of clarithromycin and hydroxychloroquine appears to have contributed significantly to the adverse neurological reaction.

Today, with increasing knowledge and experience of interstitial lung disease, the use of hydroxychloroquine in treatment is increasing. It is important to remember that hydroxychloroquine is a central nervous system stimulant, and neuropsychiatric side effects may be seen in children when used in combination with potential triggers.

## Ethics Statement

The authors have nothing to report.

## Consent

Written informed consent was obtained from the patient's guardians (parents) to publish this report following the journal's patient consent policy.

## Conflicts of Interest

The authors declare no conflicts of interest.
